# 小细胞肺癌肿瘤组织及血清CD44表达及临床预后意义

**DOI:** 10.3779/j.issn.1009-3419.2021.104.10

**Published:** 2021-08-20

**Authors:** 莹 王, 毅 郭, 海峰 林, 丽娜 张, 红梅 张, 群慧 王, 范彬 胡, 杰 李, 宝兰 李, 同梅 张

**Affiliations:** 101149 北京，首都医科大学附属北京市胸科医院，北京市结核病胸部肿瘤研究所综合科 Department of Oncology, Beijing Tuberculosis and Thoracic Tumor Research Institute, Beijing Chest Hospital, Capital Medical University, Beijing 101149, China

**Keywords:** 肺肿瘤, 干细胞标志物, CD44, 预后, Lung neoplasms, Stem cell marker, CD44, Prognosis

## Abstract

**背景与目的:**

小细胞肺癌(small cell lung cancer, SCLC)是一种以增殖迅速、易早期远处转移和获得性治疗耐药为特征的高侵袭性恶性肿瘤，临床预后极差。研究发现干细胞标志物CD44与肿瘤复发转移及治疗抵抗有关，而有关CD44在SCLC患者中的表达及其临床预后意义研究不多。本研究拟通过检测SCLC患者肿瘤组织和血清中CD44的表达水平，初步探索其与患者临床特征的关系并评估其判断预后的临床价值。

**方法:**

将本院47例初治SCLC患者肿瘤组织和血清标本配对，分别应用免疫组化法(immunohistochemistry, IHC)和酶联免疫吸附试验方法(enzyme-linked immunosorbent assay, ELISA)对CD44进行检测，分析其与患者临床特征及预后的关系。

**结果:**

SCLC患者肿瘤组织和血清中均可检测到干细胞标志物CD44的表达，体能状态(performance status, PS)2分的患者肿瘤组织CD44阳性率明显高于PS 0分-1分的患者(85.71% *vs* 30%, *P*=0.017)，根据化疗疗效对患者进行分组，疾病进展组肿瘤组织CD44免疫组化得分及血清CD44浓度均明显高于疾病控制组且差异均达到统计学意义(*P*=0.006, *P*=0.034)，单因素分析肿瘤组织CD44阳性患者无进展生存期(progression-free survival, PFS)较CD44阴性患者PFS明显缩短(5.23个月*vs* 9.03个月，*P*=0.036)。

**结论:**

初治SCLC患者肿瘤组织中CD44的表达水平与PFS相关，CD44在SCLC中的临床意义值得进一步深入研究。

## Abstract (en)

**Background and objective:**

Small cell lung cancer (SCLC) is a highly aggressive malignancy characterized by rapid growth, early metastasis and acquired therapeutic resistance, and the prognosis is extremely poor. Studies have proved that the stem cell marker CD44 is correlated with tumor recurrence and treatment resistance, however, there are limited reports yet concerning on the CD44 expression and its clinical prognostic significance in SCLC patients. The purpose of this study is to investigate the expression of CD44 in tumor tissues as well as serum of SCLC patients and explore its correlation with the clinical characteristics, therapeutic effect and prognosis.

**Methods:**

The tumor tissues and serum samples of 47 newly diagnosed SCLC patients were collected. Immunohistochemistry and enzyme-linked immunosorbent assay were applied to detect CD44. The relationship between CD44 level and the clinical characteristics as well as prognosis of the patients was analyzed.

**Results:**

The stem cell marker CD44 was detectable both in serum sample and tumor tissue of SCLC patients. The positive rate of CD44 in tumor tissue was significantly higher in patients with performance status (PS) 2 than that of patients with PS 0-1 (85.71% *vs* 30%, *P*=0.017). Patients were divided in to different groups according to the treatment efficacy. The CD44 immunohistochemical score and serum level in the disease progression group were significantly higher than those in the disease control group, and the differences were statistically significant (*P*=0.006, *P*=0.034), Univariate analysis depicted that the progression-free survival (PFS) of CD44 positive patients was significantly shorter than that of CD44 negative patients (5.23 mon *vs* 9.03 mon, *P*=0.036).

**Conclusion:**

The positive expression of CD44 in tumor tissues of pre-treatment SCLC patients is correlated with poor PFS. The clinical significance of CD44 is worthy to be further studied.

小细胞肺癌(small cell lung cancer, SCLC)是一种高度异质性神经内分泌肿瘤^[1]^，生长迅速，侵袭性强，多数患者确诊时已发生远处转移，尽管对放化疗高度敏感，多数在1年内出现复发转移，临床预后极差，局限期和广泛期SCLC患者2年生存率分别为41%和8%^[2]^。随着肿瘤靶向及免疫治疗的迅速发展，肺癌的治疗越来越个体化，总生存时间也在不断提高，然而近30年来SCLC患者生存获益改善甚微，肿瘤基础及临床研究者对SCLC关注少，用于研究的肿瘤组织有限，病理特征对临床预后的影响仍不明确，亟待寻求新的治疗策略以满足临床需求^[3]^。化疗耐药及疾病复发转移是肿瘤致死的主要原因^[4]^，揭示SCLC复发转移根源、探索并干预相关靶向标志物有可能为改善SCLC患者临床预后提供新思路。

肿瘤干细胞(cancer stem cells, CSC)学说认为，肿瘤组织中少数干细胞特性的肿瘤细胞亚群放化疗抵抗且具备自我复制及多向分化能力，与肿瘤发生、发展及疾病转归关系密切^[5]^。CD44是当前公认的SCLC干细胞标志物之一，研究发现CD44在肿瘤转移中发挥重要作用，CD44阳性肿瘤细胞相较于CD44阴性肿瘤细胞具备增殖能力强及对治疗不敏感等干细胞特性^[6, 7]^，另有体外研究及动物模型^[8-10]^证实阻断或下调细胞表面CD44蛋白表达可逆转治疗耐药及延长生存期，然而有关CD44蛋白在SCLC患者中的表达及其临床应用价值研究报道较少。SCLC患者肿瘤组织及血清中CD44表达如何，肿瘤组织中CD44的表达及血清CD44浓度能否预测患者疗效及临床预后均需进一步研究证实。本研究采用免疫组化法(immunohistochemistry, IHC)和酶联免疫吸附试验方法(enzyme-linked immunosorbent assay, ELISA)法检测初治SCLC患者基线状态肿瘤组织和血清中干细胞标志物CD44的水平，分析其与患者临床特征、治疗疗效以及预后的相关性，评价CD44在SCLC患者疗效评估和预后判断中的临床价值，以期为SCLC患者临床治疗寻求新思路。

## 1 材料和方法

### 1.1 研究设计

单中心前瞻性非干预性随访性临床研究。研究涉及的患者及其标本来自首都医科大学附属北京胸科医院肺癌队列，获首都医科大学附属北京胸科医院伦理委员会的审核批准，并获得患者或家属的知情同意。

### 1.2 研究对象

选取2017年11月-2019年12月首都医科大学附属北京胸科医院收治的病例及随访资料完整的SCLC患者。纳入标准：年龄≥18岁; 经组织病理学或细胞学确诊的在本院住院且无手术机会的SCLC患者; 体能状态(performance status, PS)≤2分或PS > 2分系因肿瘤压迫而致的急症者且能耐受一线化疗治疗者; 预计生存期≥3个月; 医生评估能耐受化疗者; 各脏器功能能耐受治疗; 依从性好，配合随访。排除标准：年龄 < 18岁; 妊娠状态的育龄妇女; 基础疾病控制欠佳，医生评估不能耐受化疗者; PS≥3分非肿瘤压迫所致者; 重要脏器如心脏、肝脏、肾脏功能存在明显异常者，治疗依从性差或有严重精神类疾病的患者。

### 1.3 试剂和仪器

主要仪器：病理染色仪Titan(福州迈新生物技术开发有限公司)，Epoch^TM^微孔板分光光度计(BIO-Tek)。一般设备包括离心管、EP管、移液器、水浴箱、去离子水、量筒、烧杯等。主要试剂：CD44人源化抗体(R & D公司，货号：AF3660)，人CD44 ELISA试剂盒(Abcam公司，货号：ab45912)。

### 1.4 血清CD44浓度测定

治疗前留取患者空腹静脉血5 mL并离心分离血清，采用ELISA法测定血清CD44蛋白浓度，操作步骤严格按试剂盒说明。酶标板每孔包被抗原，标准孔加入标准溶液，样品孔加入血清，37 ℃孵育60 min，甩干并吸净液体，每孔加入TMB(四甲基联苯胺)并室温避光孵育10 min。加入终止液，终止反应后10 min内用检测波长450 nm读值。采用Excel拟合标准曲线并根据标准曲线计算血清CD44含量。

### 1.5 肿瘤组织CD44蛋白测定

取合格的石蜡包埋组织标本常规组织切片，置于恒温箱，温度维持在65 ℃烘烤120 min取出，二甲苯脱蜡，梯度酒精脱水，去除组织内过氧化物，抗原修复，山羊血清室温封闭40 min，加入一抗4 ℃过夜孵育，次日二抗室温孵育，DAB显色，苏木素复染，盐酸酒精分化，梯度酒精及二甲苯脱蜡水化并将组织标本封片。染色强度判读：肿瘤细胞染色程度分为0分-3分：阴性为0分，弱阳性为1分，中等阳性为2分，强阳性为3分; 染色范围判读：染色阳性细胞比例计为0分-3分，0%-5%为0、5%-25%为1分，25%-50%为2分，≥50%为3分。免疫组化得分计为染色程度乘以细胞所占百分比：≤1分为阴性(-)，2分-9分为阳性，参照染色得分又可划分为3类：低表达2分-3分(+)，中表达4分-6分(++)，高表达6分-9分(+++)。

### 1.6 患者分期、治疗及随访

采用美国癌症联合委员会(American Joint Committee on Cancer, AJCC)和国际抗癌联盟(Union for International Cancer Control, UICC)第8版肿瘤原发灶-淋巴结-转移(tumor-node-metastasis, TNM)分期及美国退伍军人肺癌协会(Velterans Administration Lung Study Group, VALSG)分期标准。临床参照VALSG分期和患者体能状态给予标准的依托泊苷联合铂类化疗，局限期患者根据情况接受同步或序贯放疗。每2个周期化疗后依据第八版实体瘤疗效评估标准(Response Evaluation Criteria in Solid Tumors, RECIST)1.1将疗效判定为疾病稳定(stable disease, SD)、部分缓解(partial response, PR)、完全缓解(complete response, CR)和疾病进展(progressive disease, PD)。治疗结束后每3-4个月进行电话随访及电子病例查阅。末次随访日期2020年12月20日。

### 1.7 统计学分析

应用SPSS 22.0软件进行统计分析，根据数据分布特征，以平均数或中位数(median, M)和四分位间距(interquartile range, IQR)描述计数资料。采用卡方检验进行计数资料间比较; 采用*Kaplan-Meier*法对患者无进展生存期(progression-free survival, PFS)及总生存期(overall survival, OS)进行单因素生存分析并采用*Log-rank*检验，以*P* < 0.05为差异具有统计学意义。

## 2 结果

### 2.1 患者一般资料

本研究纳入2017年11月-2019年12月收治的90例初诊SCLC患者，最终纳入47例病理组织标本合格的患者，中位年龄62岁(35岁-80岁)，其余临床特征见表 1。

**表 1:** 47例SCLC患者肿瘤组织及血清中CD44表达和临床特征的关系(*n*=47) Correlation of CD44 in tumor tissue and serum of SCLC with patient's clinical characteristics (*n*=47)

Characteristics	*n* (%)	CD44 in tumor tissue		*P*	Serum CD44		*P*
		Positive	Negative		Higher	Lower	
Age (yr)				0.139			0.188
≥60	29 (61.70)	14	15		17	12	
< 60	18 (38.30)	4	14		2	11	
Gender				0.728			0.218
Male	39 (82.98)	15	24		22	17	
Female	8 (17.02)	3	5		2	6	
PS				0.017			0.649
0-1	40 (85.11)	12	28		21	18	
2	7 (14.89)	6	1		3	5	
Smoking history				0.879			0.378
Yes	40 (85.11)	16	24		22	18	
No	7 (14.90)	2	5		2	5	
Stage				0.345			0.629
Limited	17 (36.17)	5	12		8	9	
Extensive	30 (63.83)	13	17		16	14	
PS: performance status; SCLC: small cell lung cancer.							

### 2.2 SCLC患者肿瘤组织及血清中CD44的表达水平

SCLC患者肿瘤组织中CD44阳性率为38.30%(18/47)，其中高表达6例，中表达3例，低表达9例。CD44染色定位于细胞膜，胞浆亦可出现少量着色，可呈棕黄色颗粒，弥漫性或散在分布，胞核无着色，免疫组化染色片见图 1。

**图 1:**
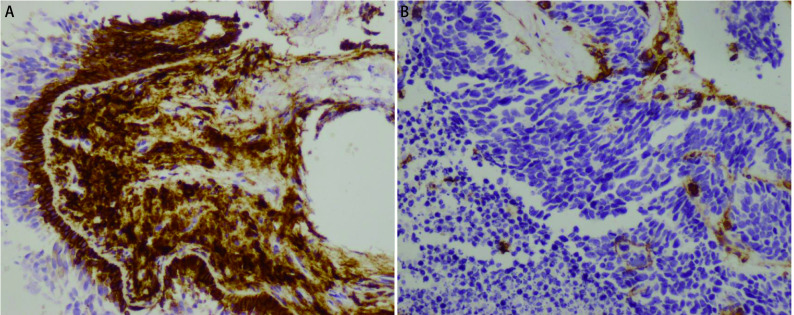
光学显微镜图片(20×)：A：CD44阳性，肿瘤细胞膜呈棕色; B：CD44阴性，肿瘤细胞膜未见染色。 Pictures of immunohistochemical staining under 20×optical microscopy. A: CD44 was positive with tumor membrane brown; B: CD44 was negative with tumor membrane not staining.

治疗前47例SCLC患者中均能检测到可溶性CD44蛋白，CD44浓度中位数111.93 ng/mL[IQR (80.88-163.63) ng/mL]。以治疗前患者血清CD44中位数为Cut-off值将患者分为CD44高浓度组(CD44≥111.93 ng/mL)和CD44低浓度组(CD44 < 111.93 ng/mL)。卡方检验分析肿瘤组织及血清CD44蛋白与患者临床特征的关系，结果显示肿瘤组织CD44阳性表达与PS评分相关，PS评分2分的患者CD44阳性率明显高于PS评分为0分-1分的患者(85.71% *vs* 30%, *P*=0.017)，结果未发现血清CD44浓度与患者临床特征有显著相关性，详见表 1。SCLC患者肿瘤组织中CD44表达与血清CD44浓度的关系应用非参数*Mann-Whitney U*检验分析肿瘤组织中CD44表达与血清CD44浓度的关系，结果发现CD44阳性表达患者相较于CD44阴性表达患者血清CD44浓度无显著差异(*P*均 > 0.05)。见表 2。

**表 2:** 47例SCLC肿瘤组织不同CD44表达分组患者血清CD44浓度比较(*n*=47) The comparison of plasma CD44 concentration in different CD44 expression groups in SCLC patients (*n*=47)

Index	*n* (%)	CD44 (ng/mL) [Median (IQR)]	*Z*	*P*
CD44 positive	18 (38.30)	113.95 (85.61-171.51)	-0.613	0.540
CD44 negative	29 (61.70)	106.89 (74.91-153.95)		

### 2.3 SCLC患者肿瘤组织及血清CD44与疗效的关系

参照化疗2周期后疗效评价结果将患者分为疾病控制组(PR+SD)和疾病进展组(PD)，因CD44免疫组化染色得分及血清CD44浓度均呈非正态性分布，采用*Mann-Whitney U*秩和检验对比不同疗效患者CD44蛋白表达水平。结果显示不同疗效分组患者CD44免疫组化染色得分及血清浓度存在明显差异，疾病进展组CD44免疫组化染色得分明显高于疾病控制组，差异有统计学意义(*Z*=-2.760, *P*=0.006)，疾病进展组血清CD44浓度明显低于疾病控制组，差异有统计学意义(*Z*=-2.123, *P*=0.034)。见表 3。

**表 3:** 47例SCLC患者不同疗效分组肿瘤组织及血清CD44表达比较(*n*=47) The comparison of tumor tissue and plasma CD44 expression level in different clinical efficiency groups in SCLC patients (*n*=47)

Clinical effects	*n* (%)	CD44 in tumor tissue [Median (IQR)]	*P*	Serum CD44 [Median (IQR)]	*P*
PR+SD	35 (74.47)	0 (0-3)	0.006	94.96 (74.21-135.83)	0.034
PD	12 (25.53)	3.5 (0-9)		137.30 (109.16-191.77)	
Result of CD44 serum concentration is expressed as ng/mL. PR: partial response; SD: stable disease; PD: progressive disease.					

### 2.4 SCLC患者肿瘤组织及血清CD44与患者预后的关系

单因素生存分析发现，肿瘤组织CD44表达阳性患者PFS为5.23个月(95%CI: 1.07-9.39)，较阴性患者的9.03个月(95%CI: 7.38-10.68)显著缩短，差异有统计学意义(*P*=0.036)(图 2A); CD44阳性患者OS为8.80个月(95%CI: 6.87-10.73)，CD44阴性患者OS为13.67个月(95%CI: 11.20-16.14)，两者相比差异虽未达到统计学意义(*P*=0.322)，但CD44阳性患者较阴性患者OS有明显缩短趋势(图 2B)。

**图 2:**
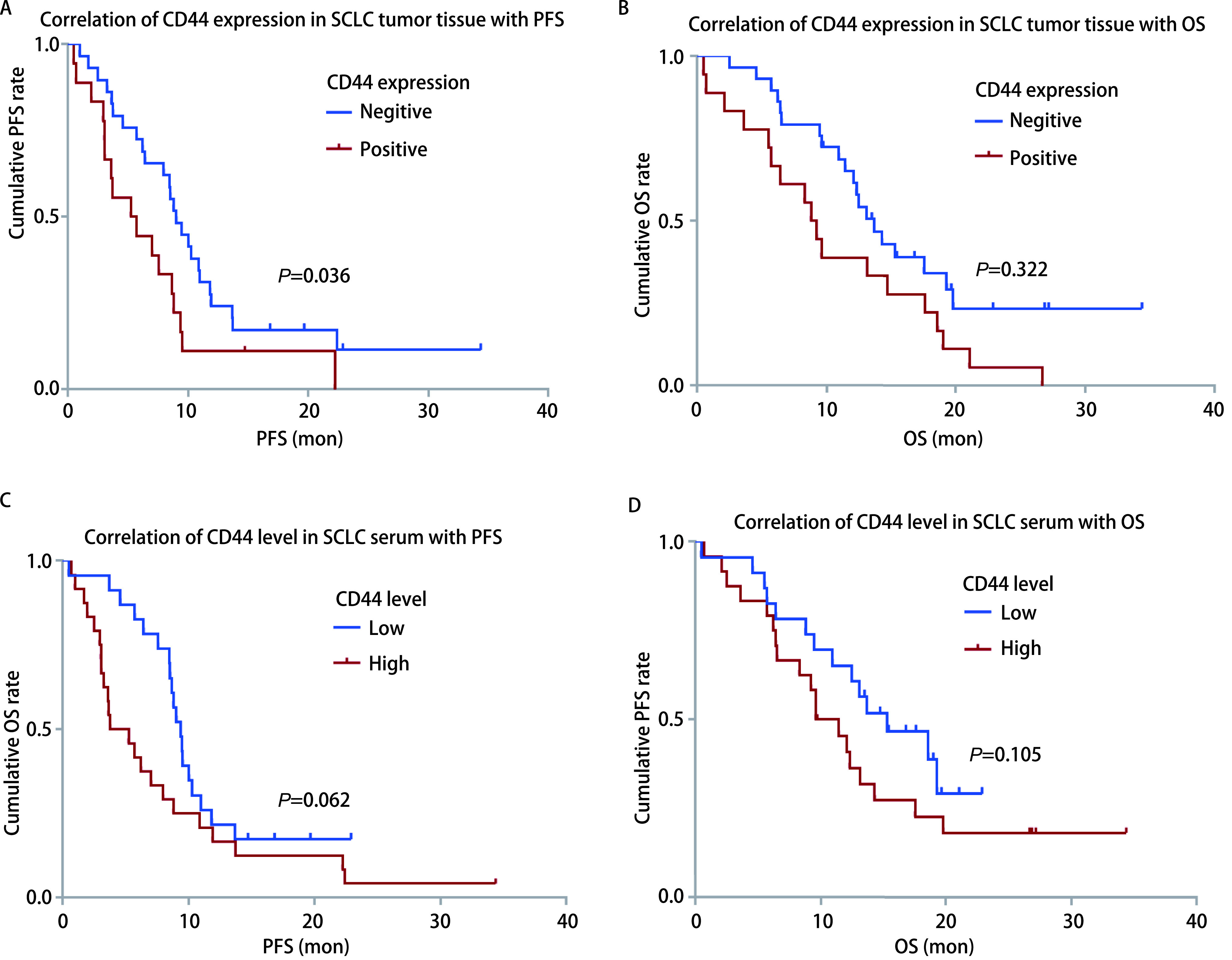
生存曲线。A：肿瘤组织CD44表达阳性患者较阴性患者的PFS显著缩短，差异有统计学意义(*P*=0.036);B：肿瘤组织CD44阳性患者较阴性患者OS相比差异虽未达到统计学意义，但存在缩短趋势(*P*=0.322);C：血清CD44高浓度组较低浓度组患者PFS差异虽然未达到统计学意义，但有明显缩短趋势(*P*=0.062);D：血清CD44高浓度组较CD44低浓度组患者的OS明显缩短，但差异未达到统计学意义(*P*=0.105)。 Survival curves. A: Patients with CD44 positive expression in tumor tissue have a significant shorter PFS compared to those with CD44 negative expression (*P*=0.036); B: Patients with CD44 positive expression in tumor tissue have a shorter OS trend compared to those with CD44 negative expression (*P*=0.322); C: Patients with high CD44 serum level have a shorter PFS trend compared to those with low CD44 level (*P*=0.062); D: Patients with high CD44 serum level have a shorter OS trend compared to those with low CD44 level (*P*=0.105).

血清CD44高浓度组患者PFS为3.77个月(95%CI: 1.25-6.29)，血清CD44低浓度组患者PFS为9.37个月(95%CI: 8.49-10.25)，两者差异虽然未达到统计学意义(*P*=0.062)，但是CD44高浓度患者较低浓度患者PFS缩短5.6个月，提示血清CD44在SCLC患者中预后评估价值有待进一步探索; CD44高浓度患者OS为13.67个月(95%CI: 9.45-17.89)，较CD44低浓度的9.60个月(95%CI: 6.34-12.86)明显缩短，但差异无统计学意义(*P*=0.105)(图 2C，图 2D)。

## 3 讨论

治疗耐药及疾病复发转移是肿瘤致死性的主要原因，但其潜在机制尚未完全阐明。研究^[6]^发现CSC治疗抵抗且增殖分化能力强，在肺癌疾病演进过程中发挥了关键作用。CD44是细胞黏附分子之一，也是公认的SCLC肿瘤干细胞标志物，可与透明质酸(hyaluronic acid, HA)结合激发细胞内信号传导途径从而诱发细胞脱落、转移、侵袭等一系列反应^[11, 12]^，另有文献^[13]^报道CD44可通过促进肿瘤细胞表面PD-L1表达来介导肿瘤增殖和免疫逃避。对干细胞标志物CD44的研究有望为肺癌临床治疗提供新的特异性靶点。

临床实践中SCLC可手术者约5%，用于研究的肿瘤组织样本不足，当前CD44在肺癌中的研究主要集中在非小细胞肺癌(non-small cell lung cancer, NSCLC)患者，SCLC患者中的研究较少且存在结论不一致的问题。Penno等^[14]^通过研究人肺癌细胞株及9例肺癌患者石蜡包埋切片中CD44发现，CD44在NSCLC肿瘤组织中高表达而在SCLC肿瘤组织表达为阴性，由此推测CD44可能是区分不同肺癌病理类型的标志物。Afify等^[15]^对52例不同病理类型肺癌的研究也指出SCLC肿瘤组织中无CD44表达。Roudi等^[16]^应用检测195例肺癌患者肿瘤标本，结果发现NSCLC患者肿瘤组织中CD44表达高于SCLC，其中肺鳞癌患者CD44表达水平最高。Gkika等^[17]^纳入98例SCLC患者的研究提示CD44在肿瘤组织表达阳性率为100%。本研究47例SCLC患者中CD44表达阳性率38.30%，与以上文献报道均存在差异。各研究结果不一致考虑与应用的CD44抗体不同相关，样本量较小及种族人群差异也会导致结论偏倚。另有文献报道SCLC肿瘤组织CD44阳性细胞少且呈散在稀疏表达，而在瘤旁及血管旁CD44阳性细胞表达增多且呈簇聚集，这些CD44阳性细胞相较于阴性细胞可能与内皮细胞及细胞外基质黏附力更强，具备更强的侵袭能力^[18]^。本研究因标本均为小活检标本，未能对瘤旁细胞CD44表达进行系统检测分析。

目前SCLC患者肿瘤组织及血清中CD44与临床特征的相关性报道尚存在一定分歧。Wimmel等^[19]^研究提示不同肺癌病理类型CD44表达差异较大，SCLC在所有肺癌病理类型中表达最低，CD44与SCLC组织病理学分级相关而与其他临床特征无关。既往有多项文献^[20, 21]^报道CD44在肿瘤组织中的表达在肺腺癌中与淋巴结转移相关。Luo等^[22]^对28项研究2, 167例NSCLC患者进行*meta*分析发现，肿瘤组织中CD44表达与肿瘤大小无关而与肿瘤分化程度、病理类型及疾病分期有关。Takigawa等^[23]^用ELISA法检测肺癌患者血清CD44s及CD44v6浓度，发现CD44s及CD44v6水平与疾病分期及淋巴结转移均无关，血清CD44v6与NSCLC病理类型有关。Shinohara等^[24]^报道则提示肺癌患者血清CD44v6浓度与吸烟及性别相关而与病理类型、淋巴结转移及疾病分期无关。我们的研究结果发现肿瘤组织CD44表达与患者PS评分相关而与其它临床特征无关，研究并未发现血清CD44与患者临床特征的相关性。各文献结论存在分歧的原因考虑因使用试剂不同、纳入研究对象不同、样本量大小及检测方法差异所致。

肿瘤组织中CD44阳性表达与治疗抵抗及不良预后的关系已在乳腺癌、胰腺癌、脑胶质瘤等多种实体瘤中得到了证实^[25-27]^，其与肺癌预后关系的研究多集中在NSCLC，且研究结果存在差异。Suda等^[28]^发现CD44可能通过上皮间质转化诱导NSCLC靶向治疗耐药，Zhang等^[29]^研究证实肺腺癌患者肿瘤组织CD44与PD-L1表达正相关，CD44高表达可提示患者预后不良，Nguyen等^[30]^则认为不同CD44亚型临床意义不同，CD44v6高表达NSCLC患者临床预后差，而CD44s与患者预后无关。但Sung等^[31]^却报道肺腺癌患者CD44高表达提示较好的临床预后。受限于可供研究的标本量不足，当前有关SCLC肿瘤组织中CD44的表达与患者预后的相关性研究较少，且亦存在结论不统一的问题，Coppola等^[32]^报道肿瘤组织中CD44表达与SCLC及肺类癌不良预后相关，而Pore等^[33]^对38例SCLC患者病理标本回顾性分析得出CD44表达与预后无关的结论。本研究单因素生存分析发现肿瘤组织CD44阳性患者PFS较CD44阴性患者显著缩短(5.23个月*vs* 9.03个月)，差异有统计学意义(*P*=0.036)，提示CD44阳性患者PFS预后差，与本研究CD44免疫组化得分高患者化疗效果差的结果一致。另结果显示肿瘤组织CD44阳性患者OS较CD44阴性患者OS减少近5个月，差异虽然未达统计学意义(*P*=0.321)，但CD44阳性患者预后有明显缩短趋势(8.80个月*vs* 13.67个月)。

有关血清CD44在肿瘤患者中的临床预后价值也是当前研究热点之一，已有文献^[34, 35]^报道多种实体肿瘤血清CD44升高，且与疾病分期及远处转移相关，是潜在的预后指标。Shinohara等^[24]^通过对261例NSCLC患者血清CD44的研究发现，血清CD44浓度高的患者预后较差。本研究结果提示疾病进展组患者血清CD44浓度显著高于疾病控制组，差异有统计学意义，与上述文献报道结论基本一致。本研究中单因素生存分析提示血清CD44浓度高的患者较血清CD44浓度低的患者PFS和OS虽差异未达统计学差异，但均有缩短趋势(PFS：3.77个月*vs* 9.37个月，*P*=0.062;OS：9.6个月*vs* 13.67个月，*P*=0.205)。血清CD44在SCLC患者中的预后判断作用值得进一步深入研究。为更全面了解CD44在SCLC患者中的临床预后意义，我们亦尝试采用生信方法获取更多信息，通过检索TCGA数据库寻找CD44在SCLC患者中的表达及其预后价值，发现TCGA数据库并未纳入SCLC数据，更加说明当前SCLC临床研究上的匮乏。

综上，本研究提示SCLC患者中肿瘤组织CD44阳性或CD44血清浓度升高可能与患者临床疗效差及不良预后相关，但由于纳入患者数量较少，尚需扩大样本进一步研究。其次，本研究筛选90例患者，最终纳入47例病理合格的患者入组，反映出SCLC患者组织病理标本珍贵，不能满足临床研究需求的现状，亟待开发新的液态活检手段以弥补不足。总之，本研究显示SCLC患者肿瘤组织及血清CD44水平与化疗疗效相关且有一定的预后判断价值，有待进一步扩大样本量及寻求新的检测方法进行深入研究，以期为SCLC临床诊疗提供新的方向和思路。
